# Advances in the application of Mxene nanoparticles in wound healing

**DOI:** 10.1186/s13036-023-00355-7

**Published:** 2023-06-08

**Authors:** Chengzhi Liang, Jing He, Yuan Cao, Guoming Liu, Chengdong Zhang, Zhiping Qi, Chuan Fu, Yanling Hu

**Affiliations:** 1 Department of Orthopedic Surgery, The Affiliated Hospital of Qingdao University, Shandong, 266000 PR China; 2 Department of Pediatric Surgery, The Affiliated Hospital of Qingdao University, Shandong, 266000 PR China; 3 Department of Orthopedic Surgery, The Second Hospital of Jilin University, Chuangchun, 130041 China; 4 Key Laboratory of Molecular Medicine and Biotherapy in the Ministry of Industry and Information Technology, Department of Biology, School of Life Science, Beijing Institute of Technology, Beijing, 100081 PR China

**Keywords:** MXene, Wound dressing, Nanoparticles, Wound healing

## Abstract

Skin is the largest organ of the human body. It plays a vital role as the body’s first barrier: stopping chemical, radiological damage and microbial invasion. The importance of skin to the human body can never be overstated. Delayed wound healing after a skin injury has become a huge challenge in healthcare. In some situations, this can have very serious and even life-threatening effects on people’s health. Various wound dressings have been developed to promote quicker wound healing, including hydrogels, gelatin sponges, films, and bandages, all work to prevent the invasion of microbial pathogens. Some of them are also packed with bioactive agents, such as antibiotics, nanoparticles, and growth factors, that help to improve the performance of the dressing it is added to. Recently, bioactive nanoparticles as the bioactive agent have become widely used in wound dressings. Among these, functional inorganic nanoparticles are favored due to their ability to effectively improve the tissue-repairing properties of biomaterials. MXene nanoparticles have attracted the interest of scholars due to their unique properties of electrical conductivity, hydrophilicity, antibacterial properties, and biocompatibility. The potential for its application is very promising as an effective functional component of wound dressings. In this paper, we will review MXene nanoparticles in skin injury repair, particularly its synthesis method, functional properties, biocompatibility, and application.

## Introduction

Cutaneous wound healing is a precise and complex process which involves multiple immune cells, non-immune cells, cytokines, growth factors, and extra cellular components [1]. The normal wound healing process can be categorized by four phases: (a) Hemostasis (b)inflammation, (c) proliferation and (d) remodeling, or three phases by considering the hemostasis/inflammation phase as one phase [2, 3]. Although these phases are interrelated, they have different processes. The first stage of wound repair are hemostasis and inflammation, which occurs immediately after tissue injury. The inflammatory pathways are thus activated, and the immune system works to remove inactivated tissue to prevent infection. It also activates the clotting cascade and promotes clotting to prevent continuous fluid loss. At the same time, the fibrin clot formed in the process of blood coagulation can be used as a barrier to prevent foreign bacteria from invading the human body through skin damage [1, 3]. Most conventional wound dressings address this first phase of skin repair in order to promote wound healing by absorbing exudate, maintaining a moist local environment, and preventing infection. The second stage of wound repair is proliferation. This process takes place between 2 and 10 days after tissue injury, and it involves angiogenesis, granulation tissue formation, and synthesis of extracellular matrix (ECM) components. In this phase, Cytokines such as vascular endothelial growth factor A (VEGFA) and fibroblast growth factor 2 (FGF2/bFGF) are secreted in large amounts in the wound, which helps to accelerate wound healing. Additionally, in this second stage, fibroblasts and myofibroblasts work together to produce extracellular matrix. Appearing as collagen, it eventually forms mature scars [4]. The third stage of wound repair is remodeling. This phase occurs around 2–3 weeks after the initial injury and lasts for about a year or more. During this stage, most macrophages and myofibroblasts will gradually apoptosis. Meanwhile, the extracellular matrix is gradually reconverted from a type III collagen backbone to a type I collagen backbone [5]. The entirety of the skin repair process shows that there are several factors involved in the process of wound healing.

**Fig. 1:**
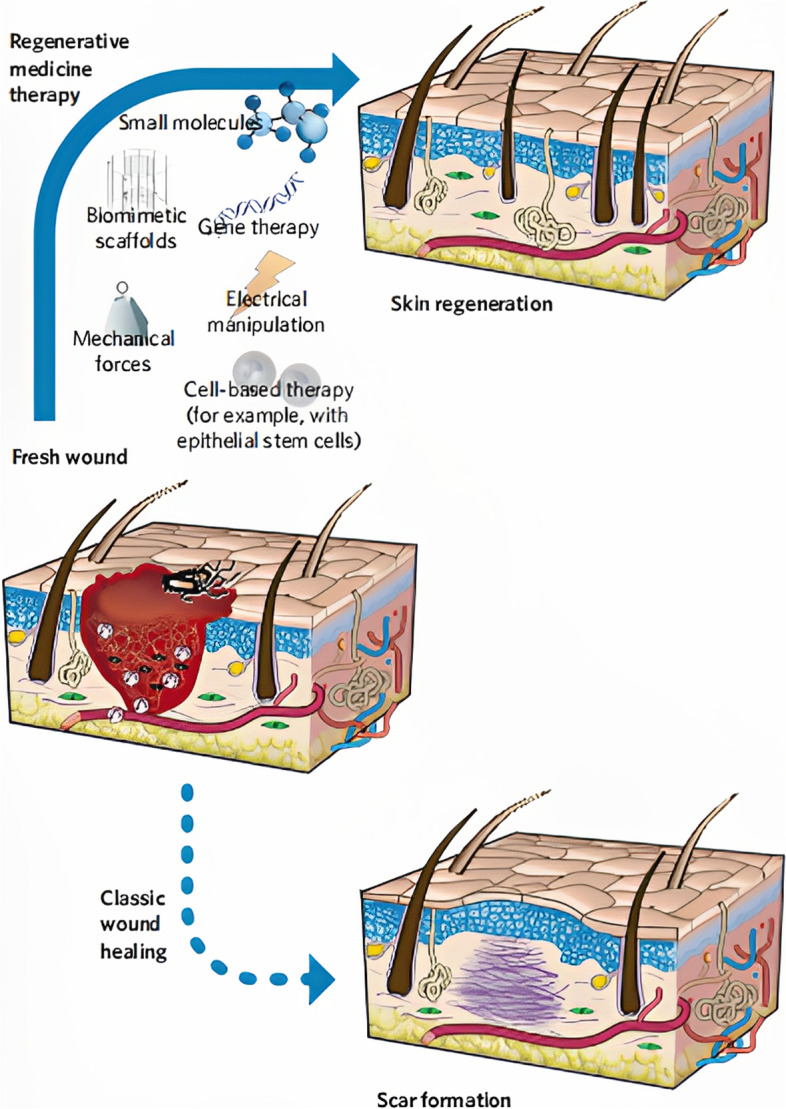
Innovative dressing strategies that promote wound healing include bionic scaffolds, electrically stimulated environments, loaded small molecules, gene therapy, and stem cells [1]

Throughout this process, many external factors can hinder the healing of the wound. Currently, infection and endogenous growth factor deficiency are considered as the two main factors that affect skin healing. The wound healing process is typically characterized by bacterial infections, which can lead to increased exudate from the wound site and can inhibit granulation tissue formation, which thereby prevents wound healing [6]. Antibiotics are widely accepted as an effective means to prevent and treat wound infections; however, the misuse and abuse of antibiotics eventually leads to bacterial resistance, which thereby reduces its therapeutic efficacy [7, 8]. Inadequate endogenous growth factors are another factor that can affecting wound healing, as they are incapable of effectively responding to local injuries, thus interfering with normal biological pathways and inflammatory responses. Ultimately, this results in delayed wound healing [9]. Traditional dressings such as gauze only play passive roles in the healing process, such as shielding, moisturizing, absorbing exudate, and preventing injury from external stimuli [10]; however, they rarely impact antimicrobial or active regulation of endogenous factors to promote wound healing. Therefore, the new dressing strategy suggests loading the traditional dressings with active substances or combining them with other therapeutic approaches to accelerate the healing process (as shown in Fig. 1). Currently, one of the most widely adopted approaches is to load dressings with active substances that have natural bactericidal properties and actively modulate endogenous related cells and factors. This method improves the antimicrobial and tissue repair capacity of the dressing [11]. A wide variety of bioactive agents have been used in the preparation of wound dressings as a way to increase their antimicrobial properties and modulate endogenous growth factor production; this is summarized in Table 1.

**Table 1:** Commonly active agents loaded in wound dressings and characteristics

Active agents	Characteristics	Reference
Ag NPs	Antibacterial properties, low toxicity, modulate anti-inflammatory, cytokine release reduced penetration into the skin.	[12, 13]
Au NPs	Antibacterial properties, low toxicity, stimulated angiogenesis and fibroblast proliferation.	[14, 15]
ZnO NPs	Antibacterial properties, low toxicity, formation of hemostatic blood clots.	[16]
Flivasorb	Absorb the exudate and retain it firmly within the dressing.	[17]
Chitosan	High bioavailability and low toxicity, antibacterial properties.	[18]
Nanocellulose	High surface area per unit, increased biocompatibility, hydrophilicity and nontoxicity, damp environment	[19]
Gelatin	Biodegradable, highly biocompatible, non-immunogenic.	[20]

**Fig. 2:**
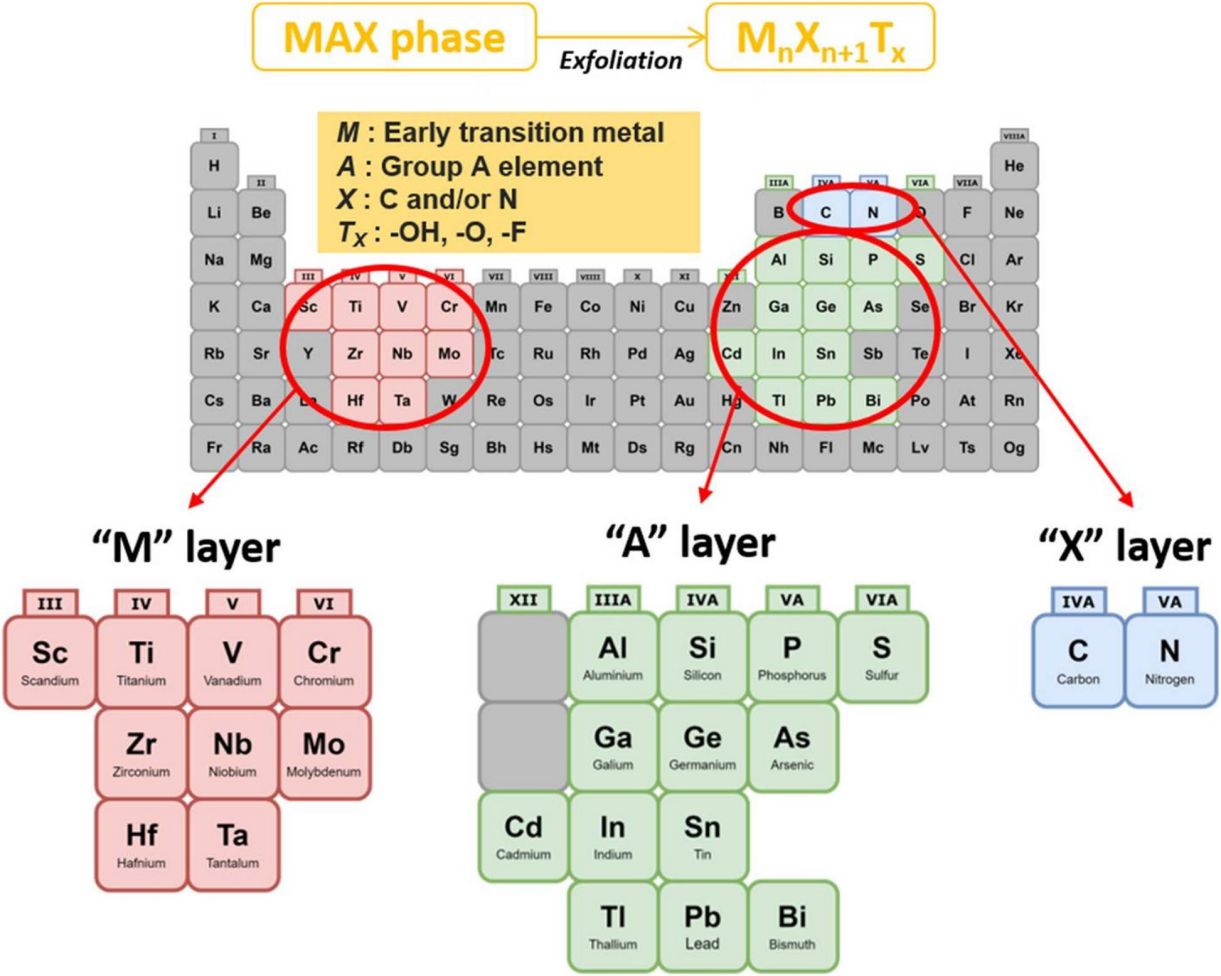
General element composition of MAX phase and MXene [28]

Among the array of active agents, MXene have come to the forefront in recent years thanks to its unique structure and physicochemical properties. MXene are a novel family of two-dimensional (2D) materials which are made up of transition metal carbides, nitrides, or carbon-nitrides [21]. They can be calculated with the general formula Mn^+ 1^Xn, where M represents transition metals (e.g., Sc, Ti, Cr, etc.) and X is carbon and/or nitrogen [22]. MXene are synthesized as a result of etching from the MAX phase. This is a layered ternary carbide and nitride with the general formula Mn^+ 1^AXn, where A represents periodic table of elements groups 13 and 14 (as shown in Fig. 2). MXene possess many unique physicochemical properties, for example: (a) MXene have hydrophilic functional groups on their surface (-OH, -O, etc.), which gives them an advantage as they do not require complex surface modification compared to other hydrophobic nanoparticles [23]; (b) MXene possess high metallic conductivity [24, 25]; and (c) MXene have excellent biocompatibility [26], allowing it to be removed and degraded in vivo [27]. Considering the above advantages, MXene have attracted extensive research interest in the field of nanomedicine. It has also been widely used in the preparation of wound dressings in recent years. In this paper, we summarized the recent developments of MXene nanoparticle composite wound dressings as well as their application prospects in wound healing. Furthermore, we analyzed their current opportunities and challenges in wound healing.

## Materials and methods

References with sturdy pages “Wound healing”, “Wound dressing”, “MXene” and nanoparticles “were indexed in PubMed and Web of science. The period of retrieval is mainly from 2015 to now.

## The synthesis of MXene

MXene synthesis can be presented as two methods: bottom-up synthesis and top-down synthesis. Choosing the suitable method for synthesis is crucial for the physicochemical properties of the synthesized products, such as the size, morphology, and function of the material [29]. The synthesized MXene can be further modified on the surface to enhance their biocompatibility for enhanced biomedical applications. Currently, the top-down synthesis from the MAX phase is the one that is most widely used.

### Top‑down approach

The top-down synthesis method has two steps: the etching of the MAX phase and the delamination of MXene. The etching of the MAX phase is the process of synthesizing MXene by wet chemical etching of the MAX phase with hydrofluoric acid (HF) or HF-containing etchant or the in situ formation of HF, like removing the A element from the MAX phase (Mn^+ 1^AXn) or the etching of Al in Ti_3_AlC_2_. In the MAX phase, there is a strong chemical bond between the A and M elements, making it difficult to remove them mechanically. Therefore, it is necessary to obtain MXene by selective etching with etchant in order to remove the ionic bonds. For the synthesis of MAX phase, Ti_3_AlC_2_ MAX phase (precursor of Ti_3_C_2_T_x_) was used as an example. Ti_3_AlC_2_ can be synthesized by putting different raw materials under different conditions. Prior to our current understanding, a mixture of Ti_2_AlC and TiC was used to synthesize Ti_3_AlC_2_ [30]. However, thanks to the development of technology, the use of cheaper raw materials (TiC, Ti, and Al) for the synthesis of Ti_3_AlC_2_ [31] emerged. Also, the mixtures of Ti, Al, and C [32], Ti, Al_4_C_3_, and C [32], and TiO_2_, Al, and C [33] have all been reported for the synthesis of Ti_3_AlC_2_. In addition to the aluminum-attached MAX phase in the above examples, some other ion-linked MAX phase precursors, such as Mn^+ 1^GaXn, Mn^+ 1^SiXn [29], can also be used for the preparation of MXene.

Firstly, etching is performed. Concentrated HF was the preferred etchant for selective removal of Al from Ti_3_AlC_2_ as reported in the literature [30]. The Max phase precursor (Ti_3_AlC_2_ powder) can be selectively etched off Al by immersing it in 50% HF at room temperature for two hours. However, high concentrations of HF have a high toxicity, which can induce cell death [29], cause systemic toxicity, and even cause death [34]. Therefore, many scholars are studying ways to effectively avoid using high concentrations of HF in the preparation process. One study compared three different concentrations of HF at 30%, 10%, and 5%. Its findings revealed that only 5% HF could effectively remove Al [31], which suggests that diluted etching reagents can be used in replacement of high concentrations of HF, in order to mitigate the toxic effects. Hydrogen fluoride salts, such as NH_4_HF_2_ [30, 35] and lithium fluoride (LiF), can be used for in situ preparation of HF for etching [24]. Although the properties of MXene prepared by in situ HF etching are similar to those of HF synthesis, it prevents the hazardous effects of HF toxicity during the synthesis process. Meanwhile, the prepared MXene is a monolayer structure, allowing for easy access to the next application.

In addition to HF etching or in situ formation of HF, it is possible to replace the “A” element in the MAX phase with other elements under high temperature conditions. For the reaction between Al-based MAX phase and ZnCl_2_ at 550 °C, Al can be replaced by Zn to obtain a Zn-based MAX phase. And when there is an excess of ZnCl_2_, Ti_2_CCl_2_ and Ti_3_C_2_Cl_2_ can be obtained. Therefore, various MXene variants can be prepared via redox reactions [36]. Studies have also reported the application of non-MAX phase precursors to prepare MXene, which can also be produced from HF treatment using layered ternary Zr_3_Al_3_C_5_ materials instead of the MAX phase as detailed here [37]. Table 2 summarizes the various methods and appropriate conditions of etching as described in this section.

**Table 2:** Methods and conditions of etching

MAX Phase	Etching solution and condition	Resulting product	Characteristic	Reference
Ti_3_AlC_2_ powder	50%HF, room temperature, 2 h	Ti_3_C_2_	High toxicity	[23]
Ti_3_AlC_2_ powder	30%HF, room temperature, 5 h 10%HF, room temperature, 18 h 5%HF, room temperature, 24 h	Ti_3_C_2_Tx	Low toxicity	[31]
Ti_3_AlC_2_ powder	1 M NH4HF2, 60℃,8 h	Ti_3_C_2_	Non-toxic, monolayer MXene	[30, 35]
Ti_3_AlC_2_ powder	LiF + 6 M HCl, 35℃,24 h	Ti_3_C_2_Tx	Non-toxic, monolayer MXene	[24]
Ti_3_AlC_2_ powder	LiF + 9 M HCl, room temperature,24 h	Ti_3_C_2_Tx	Non-toxic, monolayer MXene	[31]
Ti_3_AlC_2_ powder	ZnCl2, 550℃	Ti_2_CCl_2_ Ti_3_C_2_Cl_2_	Non-toxic, Need not MAX Phase	[36]
Zr_3_Al_3_C_5_ powder (not MAXphase)	50%HF, room temperature	Zr_3_C_2_	Non-toxic, Need not MAX Phase	[37]

**Fig. 3:**
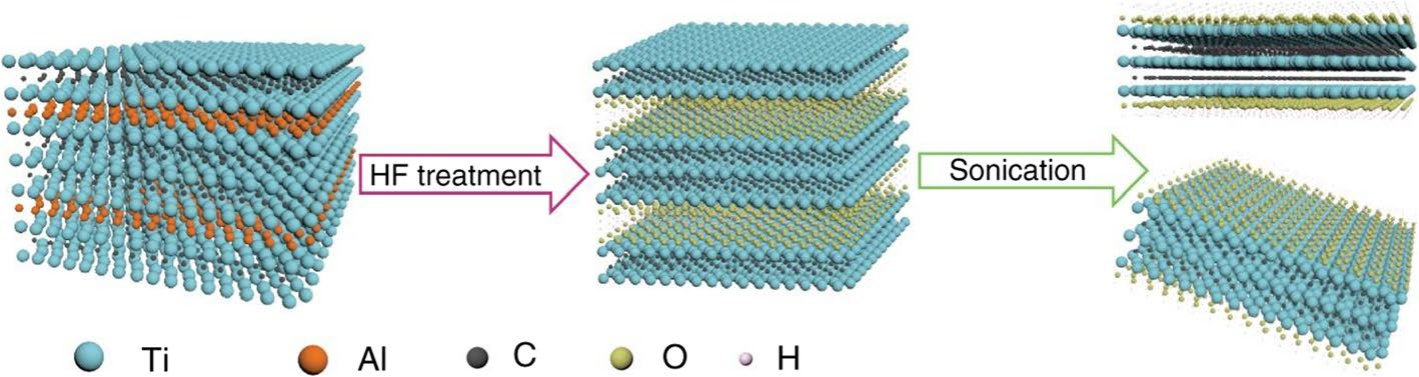
Schematic diagram of the preparation of MXene by acid etching (taking Ti3C2Tx as an example) [38]

After the selective removal of Al atoms through HF etchant, the second step is performed: delamination of MXene. The Ti_3_C_2_ layer is sonicated to terminate the top and bottom Ti layers in functional groups (Tx) such as O^2−^, OH^−^ or F^−^ (Fig. 3). However, the resulting MXene thin films often have defects which affect the material’s properties. The application of intercalating agents such as dimethyl sulfoxide (DMSO), tetrabutylammonium hydroxide (TBAOH), etc. prior to sonication can widen the interlayer spacing of MXene [23], making them easier to delaminate, and forming pure MXene thin films. Moreover, not only can intercalation compounds be used for the aforementioned purpose, it can also weaken the interactions between the 2D layers and delaminate MXene into separate 2D films. Another focus of research has been the preparation of MXene by using the minimum intensity layer delamination (MILD) method, which does not require sonication and uses a milder route to produce thin flims with larger but fewer defects [23]. An optimized MILD synthesis method using (12 M LiF/9 M HCl) at room temperature has also been reported [31]. Based on the current studies on the top-down synthesis of MXene, etchant selection, etching time, and type of intercalator are key parameters to ensure the successful synthesis of biocompatible MXene nanosheets.

### Bottom‑up approach

Bottom-up synthesis usually starts with small organic/inorganic molecular/atomic structures which undergo a crystal growth process to obtain 2D structures [23]. This has been studied [39] from the perspective of applying the chemical vapor deposition (CVD) technique to synthesize molybdenum carbide (Mo2C), which uses methane (CH4) and copper/molybdenum (Cu/Mo) foil at a temperature of 1085 °C or higher. High-quality, thin, and large nanosheets with few defects can be obtained by using this technique. However, due to the lack of functional groups on the nano-surfaces as well as the difficulty in penetrating into cells of this bottom-up method, at present, it can be deemed unsuitable for large-scale biomedical applications. In addition to CVD, existing literature details the synthesis of MXene [40] using methods such as the template method and plasma-enhanced pulsed laser deposition (PEPLD), such as 2D nitrides (MoN, V2N and W2N) [41]. Such bottom-up synthetic processes allow precise manipulation of the size distribution, morphology, and surface termination of MXene. This precision concerning the surface termination is crucial for determining the product’s properties and, consequently, its applications. Therefore, although there are few successful cases, this bottom-up approach to synthesis is still expected to be expanded in the future.

## The property of MXene

**Fig. 4:**
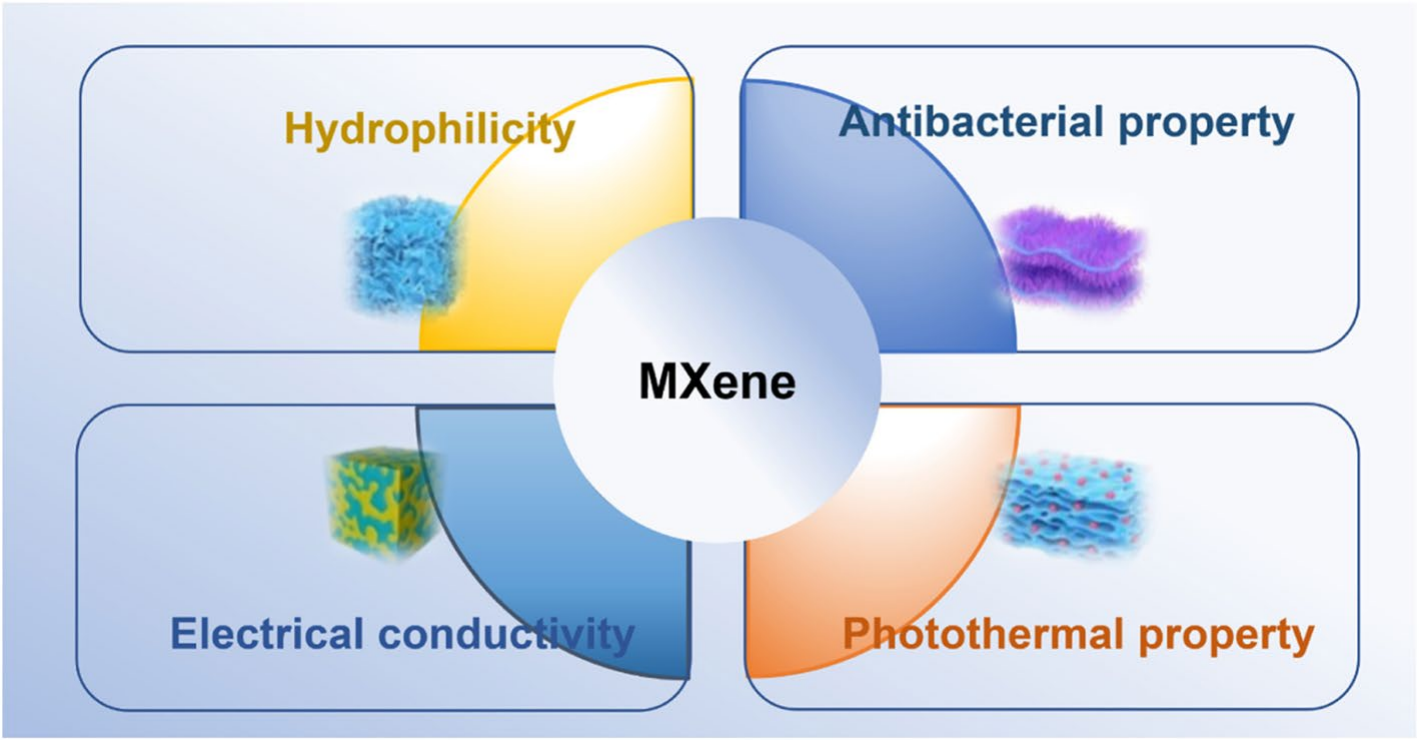
Schematic diagram of the characteristics of MXene

MXene possess a set of excellent properties, notable among which are its hydrophilicity [21, 36, 42, 43], antimicrobial [44, 45] and photothermal features [44, 46, 47], electrical conductivity [23–25, 48, 49], and good biocompatibility [26, 27]. As shown in Fig. 4, these properties are the basis for why they are highly suitable as skin repair materials. Additionally, MXene has more hydrophilic groups compared to other 2D materials, meaning they are easier to functionalize by surface modification, and, thus, easily used in biomedical applications.

### Antibacterial property

The antibacterial efficiency of Ti_3_C_2_Tx against gram-negative *Escherichia coli* and gram-positive *Bacillus subtilis* was higher than that of graphene oxide (GO), while the quantified extent of its antibacterial activity was dose dependent [44]. As revealed by SEM and TEM, in the absence of Ti_3_C_2_Tx, no cell damage or death was observed in cultured *E. coli* and *B. subtilis*. But when the Ti_3_C_2_Tx was present, varying degrees of cell damage could be seen in most bacterial cultures. It was also found the LDH release of both bacteria increased and the number of colonies decreased as the concentration of Ti3C2Tx was raised [44]. Shamsabadi et al. [45] investigated the antibacterial performance of four sizes of MXene nanosheets against *E. coli* and *B. subtilis* in dark conditions to exclude the photothermal effect of MXene nanoparticles themselves. It was discovered that the nanosheets showed bacterial DNA leakage and bacterial cell dispersion within 3 h [45]. In addition to the observational description of this antimicrobial phenomenon, Jastrzebska et al. systematically studied the structures of it [50] and the relationship between the atomic structures of Ti_2_C and Ti_3_C_2_ MXene and their antimicrobial properties. Their work yielded the conclusion that there were no differences between Ti_2_C and Ti_3_C_2_ MXene in terms of surface chemistry. However, Ti_2_C MXene did not affect bacterial survival, while Ti_3_C_2_ MXene exhibited antibacterial properties. This result was posited to be caused by the fact both have the same chemical composition at the atomic scale but different stoichiometry. In addition to inhibitory effects on commonly encountered bacteria, MXene were found to have inhibitory effects on fungi. As such, Lim et al. [51] focused upon the antifungal properties of Ti_3_C_2_Tx MXene, using inverted contrast microscopy to show how a large number of mycelium and spores were found in the control group without MXene. Conversely, fungal growth inhibition was observed in the experimental group treated with delaminated Ti_3_C_2_Tx (d-Ti_3_C_2_Tx) MXene, which led the authors to conclude d-Ti_3_C_2_Tx MXene nanosheets achieved such an outcome through disrupting the fungal hemispheric structure. Therefore, the d-Ti_3_C_2_Tx MXene nanosheets developed in this project may be a promising antifungal material which provide the basis for future research into promoting healing in infected wounds. All of the abovementioned research has shown MXene possesses good antibacterial properties and has a great deal of potential in the application of healing injuries. A considerable number of scholars have already prepared composites, with other materials serving as excipients, for the purpose of application to wounds. Table 3 summarizes the antibacterial property discussed above.

**Table 3:** Antibacterial property

MXene	Experimental method	Result	Reference
Ti3C2Tx	Observe bacterial cell damage	LDH release and the number of colonies decreased	[44]
Ti3C2Tx	Observe bacterial in dark	Bacterial DNA leakage and bacterial cell dispersion	[45]
Ti2、Ti3C2	Observe the atomic structure and antibacterial activity	Ti2C MXene did not affect the survival of bacteria while Ti3C2 MXene showed antibacterial properties	[50]
d-Ti3C2Tx	Observe the growth of fungi	Fungal growth inhibition was observed in the d-Ti3C2Tx group	[51]

### Photothermal performance

Existing literature reported Ti_3_C_2_’s properties of 100% photothermal conversion efficiency and 84% photo-water evaporation efficiency when exposed to one solar irradiation (1 kW/m^2^), which indicates MXene is a promising solar photothermal material [47]. The study also confirmed Ti_3_C_2_ nanosheets exhibit high near-infrared absorption and photothermal conversion efficiency under near-infrared laser irradiation (808 nm), as well as good biocompatibility [47]. In addition to Ti_3_C_2_, other MXene materials have similar properties, with Nb2C NS exhibiting very high photothermal conversion efficiency (36.4% at NIR-I and 45.65% at NIR-II) and high photothermal stability [27], while Ta_4_C_3_ nanosheets were found to have excellent photothermal conversion performance (efficiency η of 44.7%) [52]. All these studies indicate that, because of their excellent photothermal properties, MXene materials are a new photothermal agent with many application potential.

### Electrical conductivity

The electrical conductivity of MXene is primarily dependent upon their constituent components. Components such as Nb_2_C, Nb_2_CF_2_, etc. are metallic materials which exhibit semiconductor properties after surface modification [53, 54], and exhibit conductivity naturally. The structure of a single Ti_3_C_2_ layer is also similar to that of a semimetal which has been measured to be ≈ 0.03 µΩ m [55], and its low resistivity is a great advantage for biomedical applications. The conductivity of Ti_3_C_2_Tx can reach 6500 S cm-1 [25], and experiments demonstrated the band gap can be adjusted by changing its surface termination to improve the conductivity. Thereafter, 2D materials with high mobility and proper band gap could be obtained [56]. The conductivity of MXene has been studied extensively [23–25, 48, 49], and it has been used in applications including SMC electrodes, sensors, electronic devices, aerospace, and biomedical applications [57].

### Hydrophilicity

MXene exhibit strong hydrophilicity [29, 58–60] due to the presence of a large number of hydrophilic functional groups (e.g., hydroxyl, oxygen, or fluorine) on the material’s surface [21, 42, 43, 61]. These groups eliminate the need for complex surface modifications compared to other hydrophobic nanoparticles for biomedical applications [62]. This property, coupled with the unique surface terminations, enhance MXene’s adaptability, and those which are modified can fully exploit its properties when introduced into other composite systems such as hydrogels.

### The biocompatibility of MXene

MXene are widely used in various fields of research thanks to their unique features, but biocompatibility is a topic which cannot be avoided when exploring their biomedical applications. Biocompatibility is the most critical parameter for the evaluation of a material’s potential for biomedical use, without which there is no basic condition for application. The specific surface area and ability to self-charge make it possible to control MXene’s properties via surface modification [63]. Therefore, approaches to improve biocompatibility through surface chemistry using polymeric materials, biomolecules, or various other substances have been of interest to many scholars [64]. This section will be discussed in three parts: in vitro studies; in vivo studies; and improving biocompatibility.

### In virto

In vitro experiments have found that MXene is more toxic to cancer cell lines. An article exploring the effect of MXene (Ti_3_C_2_) on the biological activity of two normal cell lines (MRC-5 and HaCaT) and two cancerous (A549 and A375) cell lines showed optimal cytocompatibility with HaCaT. In addition, MXenes had a higher toxic effect on cancer cells compared to normal cells, while cell viability decreased with the increase of Ti_3_C_2_ concentration. The study also suggests that oxidative stress may be a potential mechanism for MXene’s toxic effects [50]. Szuplewska et al. [65] conducted an in vitro evaluation of the biocompatibility of Ti_2_NTx from four types of cells: human skin malignant melanoma cells, human immortalized keratinocytes, human breast cancer cells, and normal human mammary epithelial cells. Their experiment concluded similarly with Jastrzebska [38] that MXene is more toxic to cancer cell lines than normal cell lines. Many studies have found MXene having significant dose-dependent toxicity, Wu et al. [66] used NSCs-derived cells and primary neural stem cells to study the cytotoxicity of Ti_3_C_2_ nanosheets, TEM results showed that at a dose of 12.5 µg/ml, Ti_3_C_2_Tx nanosheets had no observed adverse reactions to NSCs or NSCs-derived cells, and when the concentration was greater than 25 µg/mL, Ti3C2 nanosheets were internalized in neural stem cells with significant cytotoxicity. Jiang et al. found that high doses of Ti_3_C_2_Tx nanosheets (> 50 µgmL − 1) produced significant cytotoxicity when treated with human mesenchymal stem cells (hMSCs) [67], and at a dose of 50 µML − 1 MXene, the onset of cell proliferation decreased after 5 days of incubation. At doses of 100 µmL − 1, hMSCs cell proliferation is significantly reduced [67]. Some studies have also suggested that MXene has no toxic effect on cells, and Zhang et al. [68] did not find significant acute cytotoxicity after treating human umbilical vein endothelial cells (HUVECs) with two different concentrations of Ti_3_C_2_Tx nanosheets (100 and 500 µgmL − 1), respectively. Breast 4T1 cancer cells and glioma U87 cancer cells were treated with PVP-1 concentrations of 0 to 200 µgmL^− 1^ with Nb_2_CTx nanosheets, and Nb_2_CTx nanosheets were found to have no toxic effects [27]. Table 3 summarizes the in vitro biocompatibility tests discussed above.

#### In vivo

Nasrallah et al. [69] used a zebrafish embryo model to assess in vivo toxicity of Ti_3_C_2_Tx nanosheets and found that Ti_3_C_2_Tx nanosheets at doses of 50 µgmL^− 1^ did not affect neuronal or muscle activity in zebrafish embryos. Alfussain et al. came to the opposite conclusion [70], using chicken embryos to explore the developmental toxicity of MXene, and experiments at a dose of 30 µg per embryo. Then they found that Ti_3_C_2_Tx nanosheets had significant toxicity to chicken embryos and observed inhibitory effects on angiogenesis in the choroid, which the analysis suggests may be caused by genes related to down-regulating cell proliferation and angiogenesis. Zhang et al. [71] implanted MXene film into the subcutaneous and calcareous defect sites of rats, and performed micro-CT evaluation and histological analysis on the samples, and the results showed that the MXene film had good bone regeneration and bone induction, and the MXene film neither induced toxic side effects nor caused inflammatory side effects. Bi_2_S_3_/Ti_3_C_2_Tx-5 prepared by Li et al. [72] exhibited excellent cytocompatibility and biocompatibility, which can promote the formation of collagen fibers and thus accelerate wound healing. At the same time, its Schottky junction also shows excellent in vivo biosafety. There were also studies that monitored the hematological parameters and biochemical index of Kunning mice after Nb2CTx injection and found no statistical significance, and the results showed that exposure to Nb2CTx nanosheets did not cause significant inflammation [27]. In addition to the above in vivo experiments, there were studies evaluating the effect of MXene on in vivo organs, Sui et al. [73] evaluated the effect of Ti3C2Tx nanosheets on organ function and its distribution in organs, and collected blood, lung, heart and other major organ specimens to determine Ti content after intravenous injection of 20 mg kg^− 1^ Ti_3_C_2_Tx nanosheets in ICR mice, and found that Ti_3_C_2_Tx nanosheets were mainly distributed in the lungs and liver. The nanosheets accumulated in the liver are gradually excreted through the bile ducts, while the nanosheets in the lungs interfere with respiratory function and lead to respiratory diseases. The above in vivo tests on MXene are summarized in Tables 4 and 5. Current research on MXene’s in vivo toxicity is still very limited, and the cytotoxicity and genotoxicity of MXene at the cellular level and the bioaccumulation and biodegradability of MXene within organs both require further research, which is essential for the further development of MXene nano-based medical application products.

**Table 4:** In vitro biocompatibility

MXenes	Type of Cells	Dose	Toxicity Effects	Reference
Ti_3_C_2_Tx	A549, MRC-5, A375, HaCaT cells	0-500 µg mL^− 1^, 24 h	Concentration dependent cytotoxicity. Toxic effects were higher against cancerous cells in comparison to normal ones.	[50]
Ti2NTx	MCF-7, A365, MCF-10 A, HaCaT cells	62.5–500 µg mL^− 1^ ,24 h	Higher toxicity to cancer cell lines than normal cell lines	[65]
Ti_3_C_2_Tx	Human umbilical vein dothelial cells (HUVECs)	100 and 500 µgmL^− 1^,48 h	No obvious acute cytotoxicity.	[68]
Ti_3_C_2_Tx	neural stem cells (NSCs) and NSCs-derived differentiated cells	12.5–100 µg mL^− 1^, 24 h	At 25 µg mL^− 1^, Ti3C2Tx nanosheets caused significant cytotoxicity to NSCs.	[66]
Ti_3_C_2_Tx	human mesenchymal stem cells (hMSCs)	0-100 µg mL^− 1^, 7 days	> 50 µg mL^− 1^, obvious cytotoxicity was shown	[67]
Nb2CTx	Breast 4T1, glioma U87 cancer cel	0–200 µg mL^− 1^, 24 h	200µgmL^− 1^, no significant cytotoiccity, exposed to NIR, cancer cells were inhibited	[27]

**Table 5:** In vitro biocompatibility

Mxenes	Type of models	Dose	Toxicity Effects	Reference
Ti_3_C_2_Tx	Zebrafish embryos	25–200 µg mL^− 1^	At the concentration of 50 µg mL^− 1^, no acutoxicity or neurotoxicity was observed.	[69]
Ti_3_C_2_Tx	Chicken embryos	30 µg per embryo,5 days incubation	Potential toxicity on the early stage of embryogenesis; down regulation of several controller genes of cell proliferation, survival, cell death and angiogenesis; inhibition of blood vessel development.	[70]
Ti_3_C_2_Tx	rats	-	No obvious inflammatory and toxic side effects	[71]
Ti_3_C_2_Tx	rats	-	excellent in vivo biosafety without damaging the main organs	[72]
Ti_3_C_2_Tx	ICR mice	20 mg kg^− 1^	Ti3C2Tx nanosheets could accumulate in the liver and lungs. Those in the lung might influence respiratory function	[73]
Nb2CTx	Kunming mice	20 mg kg^− 1^	No significant inflammation was caused. No significant histological abnormalities were found.	[27]

#### To improve the biocompatibility of MXene

In order to be able to better apply MXene nanomaterials, various methods have been used to improve the biocompatibility of MXene, and there have been studies to control toxicity through the interaction between the surface of the MXene phase and collagen [64], and surface modification has improved the biocompatibility of MXene and reduced their oxidative stress, paving the way for future nano applications. Rashid et al. [74] mapped polypropylene glycol (PPG) and polyethylene glycol (PEG) to the surface of Ti3C2Tx nanosheets and evaluated their cytotoxicity for normal (HaCaT and MCF-10 A) and cancerous (MCF-7 and A375) cell lines, a study that provides ideas for nanomaterial applications of wound dressings. Hussein et al. [75] prepared two Ti3C2Tx-based nanocomposites, Namely Au/MXene and Au/Fe3O4/MXene nanocomposites, in the human breast cancer cell line MCF7, while these nanocomposites showed similar photothermal therapeutic efficiencies, hybrid nanocomposites showed lower in vivo toxicity than pure MXene, while in vivo acute toxicity assays using zebrafish embryos indicated the use of Au/MXene and Au/A/. Fe3O4/MXene has reduced embryonic mortality [75], and this study suggests that composites can improve the biocompatibility of MXene. Wojciechowska et al. [76] used the cationic polymer poly L-lysine (PLL) molecule to alter the surface charge of Ti3C2 MXene tablets and tested the cytotoxicity of the resulting Ti3C2/PLL flakes on human skin malignant melanoma cells (A375, ATCC) and human immortal keratinocytes (HaCaT), and the results showed concentrations up to 375 mg L − 1, which did not show cytotoxicity. Similarly, Wang et al. [77] coated silkenin on an MXene film and performed cytotoxicity tests using human skin fibroblast HSAS1 cells, the silken protein-coated MXene showed about 99% cell viability after 6 days of incubation, and fluorescent image analysis showed no significant change in viability even when exposed to the silkin-coated MXene membrane HSAS1 cells, suggesting that the silkin-coated MXene film had better biocompatibility. As mentioned above, there have been considerable studies on surface modification of MXenes, and relatively satisfactory results have been achieved, but there is still a lack of in vivo tests to prove short-term and long-term safety in vivo.

### The application of MXene in wound healing

**Fig. 5:**
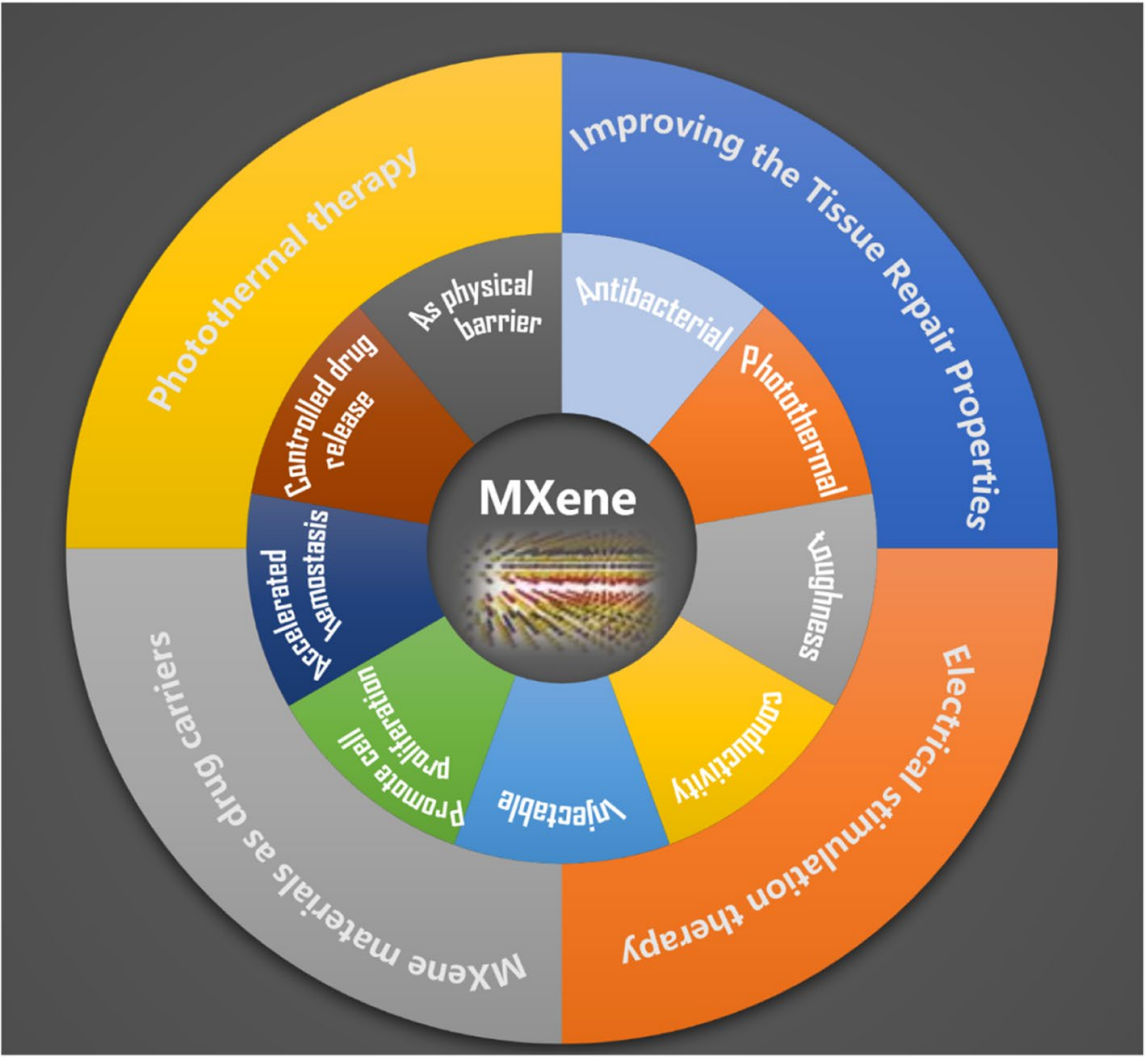
The application of mxene

At present, many scholars have developed new composite materials for the production of wound dressings, which are based on MXene nanomaterials and make full use of their excellent properties as mentioned above, and play a role in sterilization, drug-carrying sustained release, active regulation of cytokines and other functions in the skin wounds, which greatly enriches the ideas and means of treating chronic wounds. We summarize the currently retrieved MXene composites for promoting skin healing, and divide them into 4 parts according to the mechanism of action (Fig. 5).

#### Improving the tissue repair properties

Li et al. [78] prepared anisotropic MXene@PVA hydrogels by directional freezing-assisted salting out method. The hydrogel was characterized by its excellent mechanical properties (stress up to 0.5 MPa, strain up to 800%), excellent photothermal properties (localized hyperthermia can be applied to the infected site using NIR laser-808 nm), Broad-spectrum antibacterial activity (inhibition rates of Escherichia coli and Staphylococcus aureus were 98.3% and 95.5%, respectively) and promoted the proliferation of NIH-3T3 cells. In a mouse wound model, the hydrogel was found to effectively inhibit wound infection and promote Skin wound healing (98% wound closure rate). The results show that MXene@PVA hydrogel has high toughness and anisotropy, and is an excellent candidate material for wound dressings. In addition to antibacterial and pro-proliferation properties, hemostasis is also an important property for novel wound dressings. Li et al. [79] prepared a composite sponge by incorporating MXene-based nanomaterials into a chitin sponge (CH) network to achieve. The purpose of hemostasis and promotion of wound healing. After the addition of MXene-based nanomaterials, the hemostatic effect of the composite sponge was significantly enhanced due to the improvement of hemophilicity and the acceleration of blood coagulation kinetics. Moreover, the composite sponge showed excellent antibacterial activity through the synergistic effect between trapping and photothermal effects. In vivo experiments demonstrated that the wound closure rate was 84% ​​on day 9 [79]. In addition to composite hydrogels, composite sponges, etc., composite scaffold systems prepared from various materials have also been used in skin dressing research. Zhou et al. [80] combined polyglycerol-ethylene amine, Ti3C2Tx MXene@polydopamine (MXene@ PDA) nanosheets were reacted with oxidized hyaluronic acid (HCHO) to prepare HPEM scaffolds. Studies have confirmed that the scaffold has excellent rheological properties, self-healing, electrical conductivity, and tissue adhesion properties. Meanwhile, the HPEM scaffold has high antibacterial activity against Escherichia coli, Staphylococcus aureus, and MRSA, and can also stimulate cell proliferation, upregulate α muscle Actin, COL III and VEGF gene expression. Using a full-thickness MRSA-infected wound model, HPEM scaffolds were found to promote early angiogenesis in infected wounds and significantly enhance wound healing in MRSA-infected wounds [80, 81].They prepared composites by combining Ti_3_C_2_Tx MXene with antioxidant CeO_2_ and incorporated them into polyethyleneimine-grafted polyethylene ions F127 (F127-PEI) and the dynamics of oxidized sodium alginate (OSA). Formation of FOM scaffolds in Schiff-based chemically cross-linked hydrogels. This FOM stent has various functional properties, such as injectable, antibacterial, electrical conductivity, and rapid hemostasis. In vitro and in vivo experiments confirmed that the FOM scaffold could promote fibroblast migration and proliferation, granulation tissue formation, collagen deposition, and re-epithelialization to accelerate MDR-infected wound healing through electrical stimulation [81]. By combining MXene nanomaterials with various traditional materials, new composite materials such as hydrogels, composite sponges, and composite scaffolds are prepared for the preparation of wound dressings. These dressings not only exhibit the inherent properties of MXene, but also can exert the functional properties of other traditional excipients, synergistically Complementary increases the tissue repair ability of the dressing to achieve the purpose of promoting wound healing, providing a new means for wound treatment and a new idea for the research and development of new wound dressings.

### Mxene marix material-assisted photothermal therapy

Near-infrared (NIR) laser-based photothermal therapy (PTT) uses a photothermal agent as an internal energy absorber to locally convert NIR light energy into heat energy to generate high heat to cause necrosis or apoptosis of target cells. More and more scholars have discovered that photothermal therapy can be used to promote skin wound healing in addition to cancer treatment Zhu [82] et al. used the idea of ​​photothermal therapy (PTT) to construct Ag/Ti_3_C_2_Tx composites with MXene nanomaterials with excellent photothermal properties together with Ag. The antibacterial effects of Staphylococcus aureus and Gram-negative Escherichia coli were significantly enhanced compared with the application of Ag or Ti_3_C_2_Tx alone, which indicated that the antibacterial properties of Ag and Ti_3_C_2_Tx photothermal sterilization had a synergistic effect, and the cytotoxicity results showed that Ag/Ti_3_C_2_Tx had better Based on their biocompatibility, they were embedded in hydrogels for use as wound dressings and found that they exhibited excellent bacterial inhibition and wound healing-promoting properties under near-infrared light irradiation. Zhang et al. [83] prepared MXene/zeolite imidazole framework-8 (ZIF-8)/polylactic acid (PLA) composite membrane (MZ-8/PLA) by electrospinning process, which was irradiated by 808 nm laser. It exhibits effective PTT and photodynamic therapy (PDT) properties. In vitro experiments show that the antibacterial rates of MZ-8/PLA against Escherichia coli and methicillin-resistant Staphylococcus aureus are as high as 99.9% and 99.8%, respectively. In vivo experiments confirmed that MZ-8 /PLA can accelerate the healing of bacterially infected wounds without developing drug resistance.

The antimicrobial effect of photosensitive biomedical materials produces free radical oxygen (ROS) under light in addition to converting NIR to local thermal energy [84]. Li et al. [85] constructed the interface Schottky junction of Bi_2_S_3_/Ti_3_C_2_Tx, envisioning enhanced photocatalytic performance by accelerating photo-induced charge transfer to improve ROS yields for antimicrobial purposes, in vitro experiments by spread plate, live/dead fluorescence staining, and SEM antibacterial images. Other techniques have found that Bi_2_S_3_/Ti_3_C_2_Tx-5 has obvious antibacterial effects on Staphylococcus aureus and Escherichia coli, and the synergistic effect of ROS and photothermal therapy has better antibacterial properties compared with the use of ROS or photothermal therapy alone, and it was found that Bi_2_S_3_/Ti_3_C_2_Tx Schottky knot significantly promoted wound healing under 808 nm near-infrared irradiation. Similarly, Zhou et al. [86] designed and developed PLGA scaffolds with MXene as a matrix material, and Zhou et al. believe that PLGA membranes can exert a photothermal effect to generate reactive oxygen species under near-infrared light and gradually degrade to lactate, while the Lox component in the membrane can consume lactate to produce hydrogen peroxide (H_2_O_2_) and catalyze the resulting H_2_O_2_ into hydroxyl radicals through a Fenton-like reaction to achieve rapid co-sterilization. In vivo assays have shown that nanocatalytic membranes will delay the regeneration of chronic wound wounds that delay healing by antibacterial, hemostatic, local collagen deposition in wounds, and angiogenesis [86]. By producing exogenous reactive oxygen species (ROS) is a good new antibacterial concept, but due to the presence of endogenous antioxidant glutathione (GSH) in bacteria, exogenous reactive oxygen species (ROS) produced solely by light therapy may be difficult to achieve the desired antibacterial effect, Yang et al. [87] proposed a solution by preparing an antibacterial nano platform of Ti_3_C_2_/MoS_2_ bioisomer (HJ), which has photothermal, photodynamic, peroxidase-like and glutathione-like properties. Under near-infrared (NIR) laser irradiation, biological HJ not only produces local heating but also increases extracellular ROS levels, resulting in bacterial inactivation, while Mo^+ 4^ ions can invade the bacterial membrane to increase intracellular ROS content and consume intracellular GSH, and the internal and external synergistic antibacterial effects are produced. In vitro tests confirmed that biological HJ exhibited good cytocompatibility and promoted cell migration in vitro after loading FGF21. In vivo evaluation using a mouse infected wound model demonstrated that the antimicrobial platform had excellent antibacterial infection and accelerated wound healing. The above research results fully apply the photothermal properties of MXene nanomaterials, and the photothermal agent used as a composite material produces antibacterial properties and synergizes with the antibacterial properties of itself or other materials, making MXene one of the best alternative antibiotics to solve drug resistance and infected wounds.

#### MXene matrix material-assisted electrical stimulation therapy

When the epithelium is damaged, it itself generates an endogenous direct current electric field (DCEF) and a transepithelial potential (TEP) difference that drives the current out of the injured area and persists locally until the healing process is complete. Many epithelial cells, including human keratinocytes, are able to detect this electric field and make directional migration [88]. At the same time, the physiological DC electric field generated by epithelial damage can actively regulate cell behavior, enhance angiogenesis [88], block edema formation, down-regulate inflammatory factors, promote granulation tissue formation, promote collagen synthesis [89–92], and induce skin wounds. Re-epithelialization [93–95]. When the electric field was removed, wound healing slowed by 25% [88], suggesting that electrical stimulation can effectively promote skin wound healing, however, most wound dressings are not electrically active, and therefore do not have any effect on the wound site during the healing process. Responding to physiological electrical signals or external ES, which has become a major criticism of current wound dressings. To provide a physical platform for cell growth and tissue repair while also allowing localized current delivery at the wound site, Lin et al. [96] prepared bacterial cellulose/MXene (rBC/MXene) hydrogels by chemical and physical dual cross-linking method The hydrogel system can accelerate skin healing by simulating the wound healing mechanism in response to exogenous electrical stimulation (ES) according to the action of the endogenous electric field. Experiments have demonstrated that in the presence of external ES, the hydrogel and ES can synergistically enhance the proliferative activity of NIH3T3 cells in vitro and actively accelerate the wound healing process in vivo (such as reduction of wound area, enhancement of collagen synthesis and angiogenesis), It also promotes granulation tissue formation, re-epithelialization, and growth factor (including VEGF, EGF, and TGF-β) release compared with non-ES controls [96]. Overall, the biodegradable electroactive rBC/MXene hydrogel developed in this study is a promising candidate as a wound dressing for cutaneous wound healing, and this study provides evidence for promoting wound repair. An effective synergistic therapeutic strategy, which is of great significance for the development of MXene-assisted electrical stimulation therapy for wound treatment.

#### MXene materials as drug carriers for promoting skin damage repair

The use of chemical or biological agents such as drug intervention is one of the most popular methods to promote wound healing, and drug delivery systems based on different morphological designs have been proposed to prolong the drug release time and reduce the potential toxicity of drugs [97, 98]. Stimuli-responsive hydrogel systems have emerged as promising drug delivery vehicles for wound management. Yang et al. [99] developed a hydrogel system composed of MXene-encapsulated magnetic colloids and poly(N-isopropylacrylamide)-alginate double-net hydrogels to deliver photosensitizing and magnetically responsive drugs to deep chronic wounds, the results show that the hydrogel system constructed in this study has controllable drug delivery ability, which can reduce the toxic and side effects of drugs and promote wound healing. role [99]. Hao et al. [100] developed a K-M/PNIPAm hydrogel dressing based on conductive MXene nanosheets and temperature-sensitive PNIPAm polymer, and the novel hydrogel dressing was found to be strain-sensitive as well as to NIR phase transition and volume change The responsiveness of the hydrogel when used as a strain flexible sensor shows high sensitivity (gauge factor-GF is 4.491), wide strain range (about 250%), fast response speed (close to 160 ms), and good cycling stability properties (3000 s at 20% strain). Therefore, this K-M/PNIPAm hydrogel can be used as an efficient NIR light-controlled drug release carrier for on-demand drug release in deep wounds [100]. In addition to hydrogel systems, microneedle systems have also received great research interest in constructing drug delivery systems because they can penetrate the skin noninvasively and painlessly [101, 102]. Based on the above problems, Sun et al. [103] proposed 3 -(Acrylamido)phenylboronic acid-(PBA-) integrated polyethylene glycol diacrylate (PEGDA) hydrogel as host material to prepare microneedle patch, the authors believe that the derived PBA patch can not only provide exogenous adenosine It also has the ability to chelate endogenous adenosine for damage repair, and MXene can rapidly convert light into heat under near-infrared (NIR) irradiation to accelerate the release of loaded adenosine. In vitro experiments showed that MXene-integrated PBA hydrogel. The hydrogel had no negative effect on cell growth, while the adenosine-encapsulated MXene-integrated microneedle patch enhanced angiogenesis. Animal model results demonstrated that the functional microneedle patch could effectively promote angiogenesis and accelerate the wound healing process. The microneedle patch system can actively deliver and use natural molecules, which greatly enriches the variety of this type of research. Inspired by the flat and inclined structure of shark teeth, Guo et al. [104] fabricated biomimetic microneedle patches by replicating laser-engraved negative molds and using origami, which enabled the microneedle patches to have stable adhesion in tissues Adhesion, using porous ordered structures and temperature-responsive hydrogels to build controllable drug release systems on microneedle patches. Experiments in diabetic rats demonstrated that drug-loaded biomimetic microneedle patches can promote full-thickness skin wound recovery [104]. The electrospinning process has been widely used in the biomedical field. Electrospinning nanofibrous membranes have a three-dimensional network structure, which is widely studied as a promising wound healing method because it can maintain the moisture absorption balance of the wound site and promote wound healing. dressings [105]. Xu et al. [106] mixed and electrospun amoxicillin (AMX), MXene, and polyvinyl alcohol (PVA) into an antibacterial nanofibrous membrane (MXene-AMX-PVA nanofibrous membrane) for making wound dressings. In the composite nanofibrous membrane, the PVA matrix can control the release of AMX to combat bacterial infection, while the MXene can convert the near-infrared laser light into heat, resulting in localized hyperthermia to promote AMX release. At the same time, localized hyperthermia can also synergistically lead to bacterial inactivation. The antibacterial activity and wound healing ability of the composite nanofiber membrane were systematically verified in a mouse skin defect model infected with Staphylococcus aureus. The film not only functions as a co-loaded physical barrier for AMX and MXene, but also exhibits high antibacterial and accelerated wound healing abilities [106]. The drug-loading system based on MXene nanomaterials can solve the difficult problem of local drug delivery in deep tissue, and propose a new solution for tissue repair. The application of MXene in wound healing are summarized in Table 6.

**Table 6:** The application of MXene in wound healing

MXenes	Scaffold type	Result	Reference
Ti_3_C_2_Tx	composite hydrogels	High toughness, anisotropy, antibacterial activity and cell proliferation	[78]
Ti_3_C_2_Tx	composite sponge	Enhanced hemostatic effect	[79]
Ti_3_C_2_Tx	HPEM scaffolds	Excellent rheological properties, self-healing properties, electrical conductivity and tissue adhesion properties	[80]
Ti_3_C_2_Tx	FOM scaffolds	Injectable, antibacterial, conductive and quick hemostatic	[81]
Ti_3_C_2_Tx	Ag/ Ti_3_C_2_Tx composites	Bacteria inhibit and promote wound healing	[82]
Ti_3_C_2_Tx	MZ-8/PLA composite membrane	Accelerates bacterial wound healing without developing drug resistance	[83]
Ti_3_C_2_Tx	Interface Schottky junction	Antibacterial and promote wound healing	[85]
Ti_3_C_2_Tx	PLGA membranes	Antibacterial, hemostatic, promote wound local collagen deposition and promote angiogenesis	[86]
Ti_3_C_2_	Ti_3_C_2_/MoS_2_ bioheterojunction	Photothermal, photodynamic, peroxidase - like and glutathione oxidase - like properties	[87]
Ti_3_C_2_Tx	rBC/MXene hydrogels	In response to exogenous electrical stimulation	[96]
Ti_3_C_2_Tx	Double-net hydrogels	Controlled drug delivery capability	[99]
Ti_3_C_2_Tx	K-M/PNIPAm hydrogel dressing	Photocontrolled drug release ability	[100]
Ti_3_C_2_Tx	PEGDA microneedle patch	Promote angiogenesis, accelerate wound healing and active delivery	[103]
Ti_3_C_2_Tx	Biomimetic microneedle patches	Controlled drug release	[104]
Ti_3_C_2_Tx	MXene-AMX-PVA nanofibrous membrane	Physical barrier, high antibacterial	[106]

## Discussion

With adjustable surface termination, excellent performance, satisfactory properties, and suitable for a variety of biomedical applications, MXene has achieved the control of toxic side effects of the synthesis process and the properties of synthetic products by selecting the preparation method. Various composites developed based on the various properties of MXene have achieved satisfactory results in wound antibacterial and repair, showing that they are in the initial stage of development in the field of promoting skin repair and have great development prospects. At the same time, we should not only invest in developing MXene materials to promote skin healing, but also focus on finding toxicity testing solutions that meet the requirements. At present, although some scholars have conducted studies on the cytotoxicity and compatibility of related materials, these studies are based on cell experiments or short-term hematological assays, the long-term biosecurity of MXene has not been systematically evaluated, and the existing in vivo compatibility studies are carried out in low-level animal models (such as mice and zebrafish) and do not involve higher-order mammals such as monkeys or dogs, so we need to conduct more systematic MXene long-term toxicity experiments and in vivo studies of higher-level animals. This is crucial for evaluating the safety of the application of MXene materials in vivo. All in all, there are bright prospects for the study of MXene-based wound dressings and their application to clinical treatment, but we still have a long way to go.

## Data Availability

Not applicable.
